# Adhesion Optimization between Incompatible Polymers through Interfacial Engineering

**DOI:** 10.3390/polym13244273

**Published:** 2021-12-07

**Authors:** Fatemeh Mashayekhi, Julien Bardon, Stephan Westermann, Frédéric Addiego

**Affiliations:** 1 Department Materials Research and Technology (MRT), Luxembourg Institute of Science and Technology (LIST), ZAE Robert Steichen, 5 Rue Bommel, Hautcharage, L-4940 Luxembourg, Luxembourg; fatemeh.mashayekhi@list.lu (F.M.); julien.bardon@list.lu (J.B.); stephan.westermann@list.lu (S.W.); 2 Department of Physics and Materials Science, University of Luxembourg, Esch-sur-Alzette, L-4365 Luxembourg, Luxembourg

**Keywords:** adhesion, polylactide, polyimide, interfacial engineering, surface characterization

## Abstract

Additive manufacturing technologies such as fused filament fabrication (FFF) open many possibilities in terms of product functionality, including the possibility to integrate a sensor in FFF parts to perform structural health monitoring. In this context, embedding fiber Bragg grating (FBG) sensors into 3D-printed polymeric structures for strain or temperature measurements has attracted increasing attention in recent years. Indeed, offering structural health monitoring functionality can optimize the maintenance cost and increase security compared with conventional materials. However, the transmission of strain and temperature between the polymeric matrix and the FBG polymer jacket requires optimal bonding between them. In this work, the two polymers of interest are polyimide (PI) and poly(lactic acid) (PLA) for the FBG jacket and printed polymer, respectively. The current study investigates the influence of different surface treatment methods on the adhesion between a PI film and a plate of PLA, with PLA and PI being incompatible polymers. The adhesion promotion applied to the PI surface relies on cleaning, plasma activation, roughness modification, or the use of adhesive nanocoating. Bilayer samples of PI-PLA are processed by welding PLA against the treated PI by heating, whereas the adhesion between PI and PLA is measured by peel testing. It is observed that the highest adhesion between PI and PLA is achieved by a combination of mechanical abrasion increasing roughness and the use of polydopamine as an adhesive. This finding is discussed based on a synergetic effect between mechanical interlocking and chemical interaction between the two counterfaces.

## 1. Introduction

The manufacturing technology of 3D printing, and in particular FFF, is an efficient and versatile type of technology that is attractive for its many advantages over traditional polymer processes [1]. Among these promising functionalities of FFF objects, recent works reported the possibility of integrating sensors into printed objects to monitor their structural health, which was reviewed in a recent article [2].

Aside from that, polyimide (PI) is a high-performance polymer [3] widely used as a protective coating for optical fiber sensor technologies [4,5] as well as a substrate for flexible electronics [6] because of its good properties (e.g., excellent mechanical properties and thermal stability as well as good chemical resistance) [7,8]. Furthermore, PI coatings are more thermally resistant and offer good strain transfer between the sensor and measured object compared with acrylate coatings. In the last few years, fiber Bragg grating (FBG) sensors with a polyimide (PI) jacket are not only attached on the surface but also embedded into 3D printed polymeric structures during process interruptions for strain or temperature measurements. These smart 3D-printed structures allow for detecting the “inner condition” of the material. This kind of measurement is expected to be more and more prominent for high-performance printed structures, where polymers are reinforced by the incorporation of nanoparticles [9] or continuous fibers [10].

However, the functionality of these embedded polyimide FBG sensors demands an optimal bonding between the jacket and the surrounding polymeric matrix. Since polyimide is the sensor’s jacket, the adhesion between the polyimide and 3D-printed materials is particularly important for the performance of these products. There are several investigations in the literature [11,12] which have reported the successful integration of the above-mentioned sensors into additively manufactured polymeric structures. The reported deviation in readings between the FBG sensor and different strain recording devices is less than three percent [11]. Nevertheless, to date, there appears to be an absence of systematic, in-depth interfacial engineering investigations between the FBG sensor’s jacket (PI) and the host polymer matrix that shall reduce the aforementioned deviation, leading to a more precise FBG strain response and a better understanding of the relationship between the interface quality and the FBG sensor functionality.

Moreover, good bonding between the sensor and the polymeric matrix is also fundamental to improve the sensing function durability by ensuring the strain transfer at the interface. The current bonding technologies to bond two polymers or a polymer to another type of material are reported in Table 1 [13,14,15,16,17]. Bonding technologies include adhesive bonding, welding, mechanical joining, or a combination thereof. In the case of adhesive bonding, probably the most mature and versatile technology enabling the bonding of dissimilar materials, surface preparation, or activation is of primary importance to ensure high adhesion. An alternative technology is based on welding, generally requiring more complex equipment and tooling than adhesive bonding but presenting the advantage of being a very fast procedure desirable for production purposes. The bonding of dissimilar materials is an important challenge for welding technologies, requiring the development of surface preparation methods to facilitate adhesion. Concerning mechanical joining, the use of ancillary mechanical components is a mature technology, whereas joining by plastic deformation or cold forming is in its infancy for polymeric materials. In general, these bonding methods are used for engineering polymers and their composites. An important point when selecting a type of technology is to verify its suitability for utilization with thermoset or thermoplastic polymers. Indeed, the technologies are based on fusion of the polymer, as welding methods are not suitable for thermoset polymers. The joining of two thermoplastic polymers probably offers the most important technical flexibility, since these two materials can soften or melt. In addition, when thermoplastic polymers used for welding are chemically compatible, they can trigger interdiffusion and entanglement of the polymer chains across the interface upon heating, thereby providing substantial improvement of the joint quality during fusion bonding [18]. The use of adhesives is also possible for bonding two thermoplastics, especially after increasing their surface free energy by surface preparation or activation [16].

When considering the bonding between PI and most of the thermoplastic polymers, fusion bonding is partial (no melting or softening of PI occurs at the melting temperature of the thermoplastic polymer), and chain interdiffusion is not possible due to the high thermal stability of PI and PI not being compatible with the majority of the other thermoplastic polymers. The use of a conventional adhesive to bond the PI jacket of the FBG sensor to the host matrix is not desirable because it would create a thick and rigid layer around the sensor, preventing proper operation. Nevertheless, in [19], the bonding of PI to polydimethylsiloxane (PDMS) was explored by means of an epoxy adhesive reaching a low peel strength of 0.0017 ± 0.0009 N/mm. The latter was drastically increased by combining the adhesive with surface functionalization, enabling it to reach a maximum peel strength of 0.46 ± 0.04 N/mm. By only applying surface functionalization, the peel strength of the PI-PDMS samples reached a maximum of 0.20 ± 0.04 N/mm. Hence, interfacial engineering appears to be highly desirable for bonding PI to another polymer, which would require further investigations.

**Table 1:** Main bonding technologies to bond two polymers or a polymer to another material (non-exhaustive information), based on the reviews [13,14,15,16,17] (Copyright (2021), with permission from Springer Nature).

Bonding Type	Bonding Method	Surface Preparation or Activation Method	Application
Adhesive bonding	Chemically reactive adhesive (epoxy, toughened acrylic) Moisture cure adhesive (polyurethane, silicone) Anaerobic adhesive (anaerobic acrylic) Hot melt adhesive (polyamide-based adhesive) Light cure adhesive (UV-curing acrylic) Heat cure adhesive (single component epoxy adhesive) Pressure sensitive adhesive	Chemical cleaning (solvent cleaning and degreasing, detergent cleaning, alkaline cleaning) Mechanical treatment (abrasion) Surface modification (etching or oxidation, sodium treatment, iodine treatment, surface grafting, thermal treatment, primers) Electrical discharge treatments (corona discharge, plasma discharge) Other treatments (laser, transcrystalline growth, ultraviolet radiation, flame treatment, removal of surface layers)	Polymers and polymer-based composites for engineering applications (aerospace, automotive, medical, packaging, clothing, ballistic protection, electronics)
Welding	Thermal stress (laser, hot press, hot gas, extrusion, infrared) Friction or viscoelastic deformation (friction stir welding, vibration, spinning, ultrasonic) Electromagnetic field (resistance, induction, microwaves, radiofrequency)		
Mechanical joining	Ancillary mechanical components (screw, rivet, pin) Plastic deformation or cold forming to join two materials	Chemical cleaning	

In the current study, the improvement in adhesion strength between PLA and PI is investigated through different methods applied on model materials. PLA is a popular thermoplastic polymer for FFF applications due to its attractive properties, such as its low shrinkage, low processing temperature, and moderate cost [20]. The studied treatments include (1) PI surface preparation by cleaning, plasma activation, and roughness modification by abrasion, (2) adhesive bonding by the deposition of an adhesive nanocoating on PI, and (3) the welding of PLA by heating against PI. The use of a nanocoating adhesive such as polydopamine (PDA), known as a universal adhesive [21], is expected to better transmit the thermoplastic matrix strain level to the sensor [22,23], compared with the use of a conventional thick adhesive (Table 1) that may block or dissipate strain [24]. Aside from that, PDA is deposited from an aqueous solution of dopamine (DA), thereby preventing the utilization of hazardous solvents. Moreover, PDA nanocoating can be deposited on a wide range of materials, and its deposition kinetics is not affected by the substrate’s surface chemistry [23]. The potential chemical interaction between PI-PLA, PI-PDA, and PLA-PDA is preliminarily estimated based on the Hansen solubility parameters (HSP) [25]. After conducting various surface treatments, the adhesion between PI and PLA is measured by peeling testing. The underlying adhesion mechanisms are hypothesized from the results of different surface characterization methods.

## 2. Materials and Processing

The selected polyimide material was the reference Thermalimide procured from Airtech Advanced Materials Group (Differdange, Luxembourg) as an amber transparent film with a thickness of 50 µm. The PI film, with a width of about 1 m and a length of about 6.5 m, was received in roll form and packed in a plastic bag. Both surfaces of the film were uncoated (no release agent present at the surface). According to the supplier, the maximum use temperature of this PI film is 426 °C, indicating a very high thermal stability [26]. PLA pellets were supplied by NatureWorks (Minnetonka, MN, USA) under the reference 4043D.

For the adhesion experiment, PI films of dimensions 100 mm × 100 mm × 50 µm were cut from the PI bagging sheet roll, and the polylactide (PLA) pellets were compression-molded using a hydraulic press (Labtech Engineering Co. Ltd., Bangpoo, Thailand) to make plates 100 mm wide, 100 mm long, and 2 mm thick. Before processing, the PLA pellets were dried for at least 24 h at 50 °C and under a vacuum in a Thermo Scientific Heraeus (Langenselbold, Germany) oven. After loading the mold, each sample was preheated at 200 °C for 2 min to permit melting. Subsequently, while the temperature was still 200 °C, a pressure of approximately 40 bar was applied for 4 min to melt the polymer and hence homogeneously fill the mold. Before removing the samples, the press plates were water-cooled under pressure to room temperature. The PLA plates were thereupon cleaned with isopropyl alcohol in an ultrasonic bath for 30 min and then dried and kept at 50 °C in a vacuum oven (Thermo Scientific Heraeus, Langenselbold, Germany) before conducting the compression molding with the PI film.

Bilayer samples of the treated PI film and PLA plate for peel testing were put into close contact and prepared by a procedure identical to the plate preparation, except that neither preheating nor external pressure were applied. This procedure enabled the welding of PLA against PI by heating.

## 3. Estimation of Solubility Parameters

Comparing the cohesion energy parameters or Hansen solubility parameters (HSP) of two substances allows the prediction of their chemical affinities, solubility, and solubility-related phenomena. The chemical compatibility or cohesion between two substances will reach a maximum when the HSP match, which has a significant impact on the interface properties. In the current study, to have an estimate of the adhesion behavior of PI on the PLA, the affinity between PI and the PLA is calculated based on the Hansen solubility parameters theory and using the Hansen Solubility Parameters in Practice software (HSPiP, Steven Abbott and Hiroshi Yamamoto, version 5.0.13 x64) [27].

The polymer’s chemical structure was converted into a string of symbols, the “Simplified Molecular Input Line Entry Specification” (SMILES), and was entered as the input for chemical calculations in the HSPiP software. Then, the HSP (δ_D_, δ_P_, and δ_H_) were determined for PI, PLA, and dopamine by the software, which corresponded to the substance’s sphere center in Hansen space. The knowledge of HSP enabled the calculation of the spatial distance R_a_ between the HSP of the two polymers based on the following equation [25]:

R_a_ = [4(δ_D PI_ − δ_D PLA_)^2^ + (δ_P PI_ − δ_P PLA_)^2^ + (δ_H PI_ − δ_H PLA_)^2^]^0.5^ (1)

where δ_D_, δ_P_, and δ_H_ were the cohesion energy from the dispersion forces, polar forces, and hydrogen bonding, respectively, calculated for PLA (δ_D PLA_, δ_P PLA_, and δ_H PLA_) and PI (δ_D PI_, δ_P PI_, and δ_H PI_). Finally, the relative energy difference (RED) number was calculated using the formula below [25]:

RED = R_a_/R_o_ (2)

where R_o_ is the PLA sphere radius, which was considered to be 10.7 (MJ·m^−3^)^0.5^ [28]. The closer the RED value was to zero, the more compatible the two polymers were, and the more affinity they had for each other. The value of one is theoretically the soluble/insoluble border. The calculated RED number and HSP of PLA and PI are reported in Table 2. The RED number of PI-PLA was 1.05, predicting that these two polymers were not compatible.

The RED number and HSP of dopamine used as an adhesive in the current experiment were also calculated in the same way, and they are displayed in Table 3. The RED numbers of DA–PI (0.58) (R_o PI_ = 21.6 (MJ·m^−3^)^0.5^ [23]) and DA–PLA (0.60) were lower compared with that of PI–PLA (1.05), showing that according to this estimation, DA was expected to be compatible with both PI and PLA and hence could be proposed as an adhesive.

## 4. Methods

### 4.1. Surface Treatments

A variety of surface treatment methods were considered to maximize the adhesion between PI and PLA. These methods are listed in Table 4 and include (1) PI film cleaning in a solvent (followed by drying), (2) PI film grinding with a polishing paper (mechanical treatment by abrasion), (3) deposition of a nanocoating of polydopamine (PDA) adhesive onto PI, (4) atmospheric plasma activation of the PI surface to finely clean and chemically modify the surface, (5) chemical etching of PI to clean its surface, modify its chemistry, and potentially increase the roughness in the case of preferential etching of some molecular sections of the structure, and (6) a combination of treatments (2) and (3). Overall, 6 different cases were considered (Table 4) to evaluate the effectiveness of each treatment. As indicated in Table 4, these different strategies were expected to play on the surface wettability (by cleaning or chemical modification of the surface) and surface roughness to create mechanical interlocking between the rough PI surface and the melted PLA and increase the chemical interaction through a coating between the coated PI and PLA. Peel testing was conducted to evaluate the adhesion strength between PI and PLA, whereas surface characterizations were conducted to identify the underlying mechanisms. These characterizations included contact angle (CA) measurements, surface free energy (SE) calculations, and atomic force microscopy (AFM).

#### 4.1.1. Cleaning

The PI film was cleaned with isopropyl alcohol of a purity ≥99.5% (Fisher Chemical, New Hampshire, NE, USA) in an ultrasonic bath for 30 min and then dried at 50 °C under a vacuum overnight to remove unnecessary moisture using the Thermo Scientific Heraeus (Langenselbold, Germany) vacuum oven. Note that the cleaned PI film was systematically kept at 50 °C in the vacuum oven before conducting the next treatment or the compression molding with PLA to lower the moisture recovery and slow down surface re-contamination.

#### 4.1.2. Mechanical Abrasion

The PI film was ground manually in the vertical and horizontal directions for 3 min in total with silicon carbide grinding paper of FEPA P #2000 (grain size of 10 µm) (Struers, Ballerup, Denmark) and water as a lubricant. As described in Table 4, the grinding step was always followed by a cleaning step to remove grinding residues from the PI surface.

#### 4.1.3. Plasma Treatment

The PI film surface was treated with an atmospheric dielectric barrier discharge (DBD) plasma reactor [29]. The plasma power and frequency were set to 450 W and 6 kHz, respectively. The working gas was a mixture of 80 vol% of nitrogen and 20 vol% of oxygen, whereas the total gas flow was set to 20 L/min. The samples were positioned on the bottom electrode and exposed to plasma when the high-voltage top electrodes were moved back and forth over the samples. The number of movements (passes), movement stroke, and top electrode speed were set to 8, 0.4 m, and 4 m/min, respectively, which determined the total treatment time (i.e., 48 s).

#### 4.1.4. Adhesive Nanocoating

A 1.2-g/L Tris(hydroxymethyl)aminomethane (Euromedex, Strasbourg, France) water buffer solution was first prepared to obtain a pH of 8.5, and 2 g of dopamine hydrochloride (Sigma-Aldrich, Steinheim, Germany) per liter was then dissolved in the buffer solution. Following that, the PI film was immersed in the dopamine solution for 24 h. Note that the reaction solution was continuously stirred (200 rpm) at room temperature (21 °C ± 2 °C). An ultra-thin polydopamine (PDA) layer was expected to adhere to the samples’ surface via the oxidative self-polymerization of dopamine, as described in previous papers [30,31,32]. Then, the PDA-coated samples were taken out from the reactive solution, washed with deionized water, and dried in the vacuum oven at 50 °C overnight.

#### 4.1.5. Chemical Etching

This treatment was carried out on PI to increase the surface wettability. The effect of two etchants, sodium hydroxide (NaOH) (Carl Roth, Karlsruhe, Germany), and hydrochloric acid fuming 37% (HCl) (Carl Roth, Karlsruhe, Germany) was investigated by varying the etching duration. The PI samples were etched in a 3N NaOH solution at 40 °C [33] and HCl solution at room temperature for different durations and then washed thoroughly in distilled water and dried. The PI films were etched with NaOH for 15 min, 30 min, 1 h, and 2 h, yet the films etched for more than 30 min were warped after drying, and hence, only the PI film etched for 30 min was used for the adhesion experiment.

## 5. Surface Characterization

### 5.1. Contact Angle and Surface Free Energy Measurements

The wettability of solids by liquids is influenced significantly by their surface free energy. As a result, this energy is an important parameter for optimizing the solid PI and molten PLA contact during bilayer sample preparation of the PI film and PLA plate. The PI film wettability before and after surface treatment was investigated via static contact angle measurements. The surface energy of the PI film was determined by measuring the contact angle of the distilled water and diiodomethane on treated surfaces and through use of the Owens, Wendt, Rabel, and Kaelble (OWRK) method to evaluate the surface energy characteristic values [34,35].

The sessile drop technique was used to measure the contact angles with a Dataphysics OCA 15 contact angle device (DataPhysics Instruments GmbH, Filderstadt, Germany). In the case of the water and diiodomethane contact angle measurements, by means of a syringe pump, a 2-µL and 1-µL sessile droplet, respectively, were deposited at the surface of the treated PI films, and the contact angle value was calculated from the captured image of the droplet shape using the Laplace–Young model. The average value of the contact angle was calculated from 10 droplets, which were deposited at various positions on the of treated PI’s surface.

### 5.2. Atomic Force Microscopy

Changes in the surface roughness of the PI film before and after surface treatments were measured with an Innova atomic force microscope (AFM) (Bruker, Santa Barbara, CA, USA). The images were acquired in tapping mode at scan rates between 0.5 Hz and 2 Hz. Silicon OPUS 160AC-NA AFM tips (Mikromasch, Bulgaria) with an average cantilever spring constant of 26 N.m^−1^ were used. Three images of 10 µm × 10 μm and 1 µm × 1 μm were recorded for each treated PI film. Each image was corrected for tilting, and the roughness parameter values were calculated using MountainSPIP software (Digital Surf, Besançon, France). The arithmetical mean height (Sa) value of the surfaces was reported as a surface roughness amplitude parameter.

### 5.3. X-ray Photoelectron Spectroscopy

The surface chemistry of the materials was characterized by X-ray photoelectron spectroscopy (XPS) utilizing a Kratos Axis Ultra DLD photoelectron spectrometer (Manchester, England). This analysis was performed with monochromatic Al Kα radiation (1486.6 eV) and an X-ray power of 150 W. With the samples being insulating, a charge neutralizer was used, leading to an energy resolution of 0.9 eV for the narrow scans. The area of analysis was set to 700 µm × 300 µm, and all the measurements were performed with a 90° take-off angle. The analysis depth was about 10 nm. The data were processed with CasaXPS software (version 2.3.22). The binding energy scale of all the spectra was corrected by placing the main C-(C, H) components at 284.9 eV. The elemental composition was obtained by using the relative sensitivity factors from the spectrometer’s library, and the peak fits of the spectra were performed with symmetrical Gaussian-Lorentzian (70–30) components.

## 6. Adhesion Measurements

**Figure 1:**
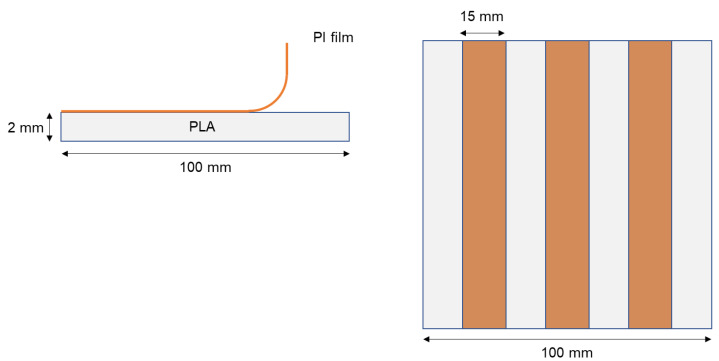
Schematic of the peel testing sample showing the side (**left**) and top view (**right**).

The adhesion between the treated PI film and PLA plate (Bilayer sample) was evaluated using 90-degree peel testing. Three PI stripes with a width of 15 mm were cut in the bilayer samples, avoiding the lateral edge effect (Figure 1). The unbonded end of the flexible PI film was gripped in the test machine jaw. The peel strength (i.e., the average load per unit width of the bond line (15 mm)) to separate the treated PI film from the PLA substrate was measured at a crosshead speed of 250 mm/min using an Instron universal testing machine 5967 (Norwood, MA, USA) equipped with a load cell of 100 N. Note that the first 25 mm of peeling were disregarded for the peel strength calculation based on the standard ASTM D6862-11.

## 7. Results and Discussion

### 7.1. Contact Angle and Surface Free Energy Measurements

**Figure 2:**
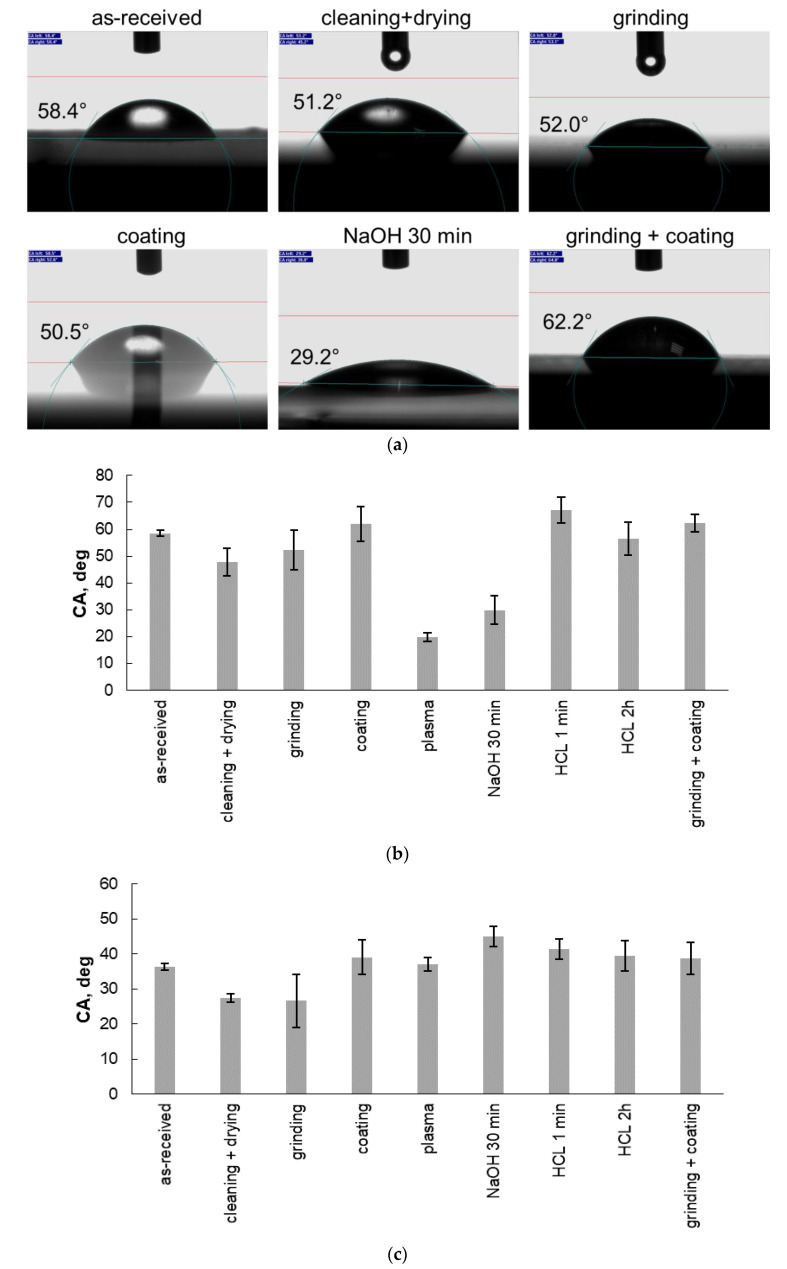

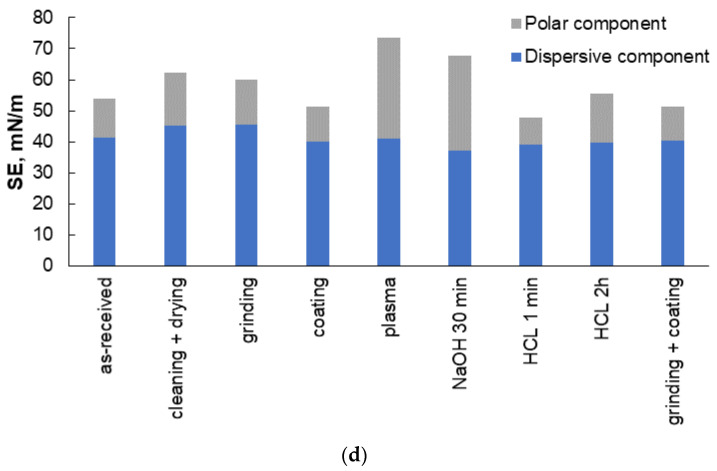
(**a**) Selected water droplet profiles and water contact angle evaluation; (**b**) Average water contact angles of the PI films; (**c**) Average diiodomethane contact angles of the PI films (the standard deviation calculated from 10 tests for each treated PI film is represented by the error bars); (**d**) Total surface free energy (SE) (with its polar and dispersive components) of the PI films before and after the different surface treatment methods.

Some selected water droplet profiles and the average results of 10 contact angle (CA) measurements with water and diiodomethane are shown in Figure 2a–c for the different surface treatments. The surface free energy (SE) values of the studied cases are reported in Figure 2d. All the surface treatments modified the contact angle and the surface free energy of PI. All the treatments, except PDA coating deposition and etching by HCl for 1 min, increased the free surface energy and hence the wettability of the PI surface. The highest increase in the free surface energy was obtained by plasma treatment (73.6 mN/m vs. 54.0 mN/m) for as-received PI. Nevertheless, all the cases exhibited a free surface energy higher than 40.0 mN/m, and hence, the PI surfaces exhibited a rather high wettability compared with other polymers [36]. In the case of the PLA plate, contact angle measurements were also conducted after the same cleaning procedure as that for the PI film, enabling a surface free energy of 48.3 mN/m (9.8 mN/m for the polar component and 38.5 mN/m for the dispersive component) to be obtained. In theory, to have good wetting of PLA on PI, and assuming the PLA is not changing its surface energy upon melting, the surface free energy of the PI should be higher compared with that of PLA, indicating that PI after plasma treatment shall exhibit the best wettability among all the treatments.

### 7.2. Adhesion Measurements

**Figure 3:**
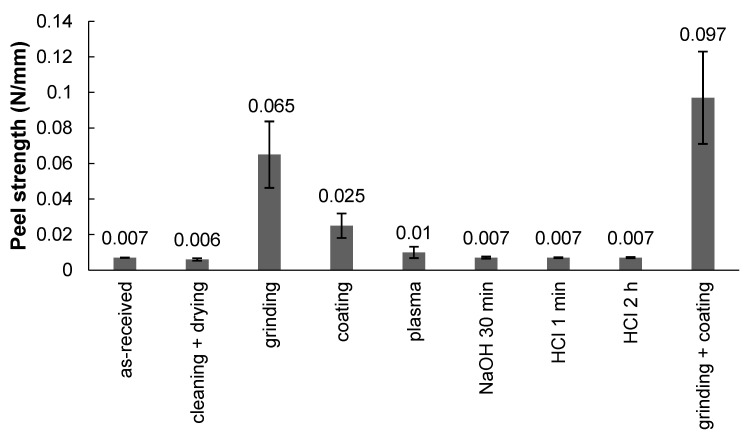
The peel strength of the bilayer samples.

The peel strength of the bilayer samples required separating the treated PI films as shown in Figure 3. The reference PI (as-received film)-PLA samples had a peel strength of 0.007 ± 0.0001 N/mm. The peel strength was higher for the grinding + cleaning + drying + PDA coating case (0.097 ± 0.0260 N/mm), which was a combination of two effects: grinding and PDA coating. The second-best result was observed for the grinding + cleaning + drying case (0.065 ± 0.0187 N/mm). Therefore, it can be noted that when comparing these two best cases, grinding provided a higher increase in peel strength compared with the other used surface treatment methods. The treatment of cleaning + drying + PDA coating exhibited a peel strength of 0.025 ± 0.0069 N/mm (i.e., a significant improvement compared with the untreated (cleaned) sample, but lower than the case of grinding + cleaning + drying (0.065 ± 0.0187 N/mm)). This finding proves that the increased wettability recorded by the contact angle measurements (Figure 2) in the case of cleaning, chemical etching, and plasma treatments was not correlated with the large increase in adhesion between PI and PLA. In the following sections, characterizations are conducted in the cases providing the highest adhesion for a better understanding of the underlying mechanisms.

### 7.3. Atomic Force Microscopy

**Figure 4:**
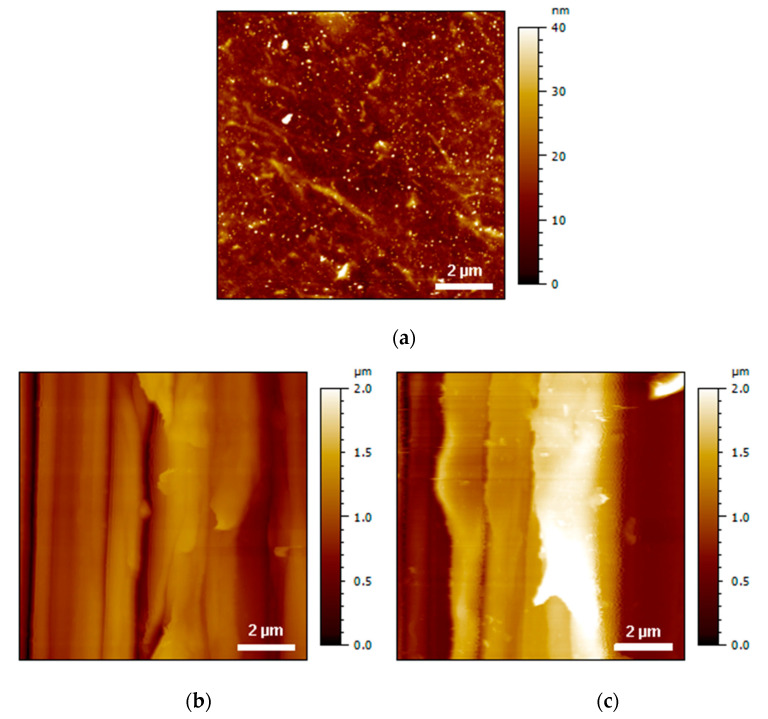
AFM 2D height images of PI film (**a**) before and (**b**) after grinding + cleaning + drying surface treatment and (**c**) after grinding + cleaning + drying + PDA coating surface treatment.

**Figure 5:**
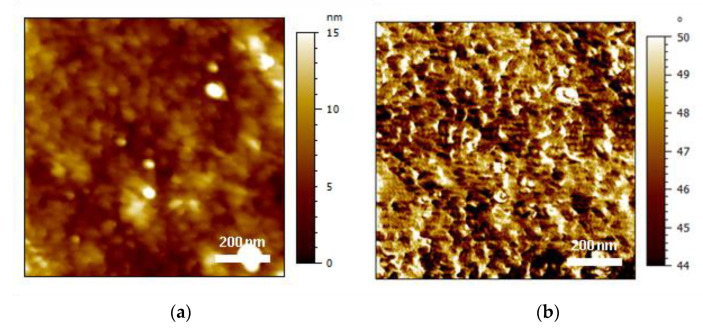

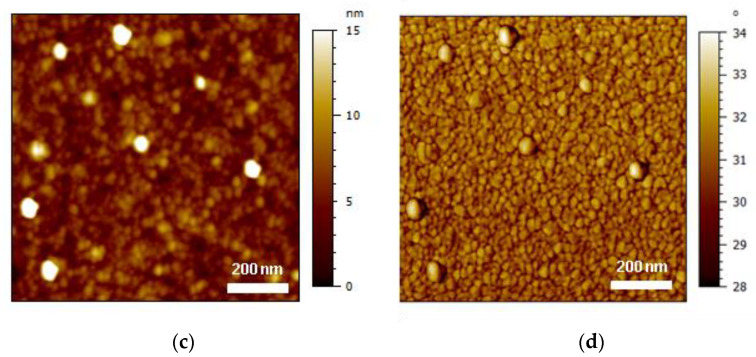
AFM images of PI film topography (**a**) and phase (**b**) on bare PI, as well as topography (**c**) and phase (**d**) after PDA coating.

The AFM analysis was conducted at two scales, which were areas of 1 µm × 1 µm and 10 µm × 10 µm, to obtain information about the topography at two different scales. Indeed, grinding by mechanical abrasion drastically modified the material surface compared with the untreated materials. The average roughness amplitude (Sa) of the different cases of interest is listed in Table 5, whereas some AFM height images are presented in Figure 4 and Figure 5 for the two scales of interest. After PDA coating, the roughness increased from 4.04 ± 0.73 nm to 6.95 ± 1.98 nm in the case of the large-scale images (Figure 4). In the case of the small-scale images (Figure 5), no significant effect of the PDA coating on the roughness was observed. After grinding, both scales exhibited a drastic increase in the roughness amplitude, with the latter being more marked in the case of the largest areas. When combining PDA with grinding, an increase in roughness was detected for both areas, although an important deviation of the data was noted. The AFM image reveals that the surface roughness was drastically increased by both grinding and PDA coating, leading to grinding or wear tracks. A comparison of the AFM 2D height and phase images of the PI film before and after PDA coating (Figure 5b,d) clearly shows a more marked aggregate morphology on the PI-coated surface, which is the first information about the morphology of the PDA coating. The PDA aggregates varied in size (mainly in the nanometer range) and homogeneously covered the surface of the PI, confirming previous studies [37]. The particulate shapes at the sub-micron scale are observed in Figure 4c, and it was assumed that these objects were large PDA aggregates.

### 7.4. X-ray Photoelectron Spectroscopy

**Figure 6:**
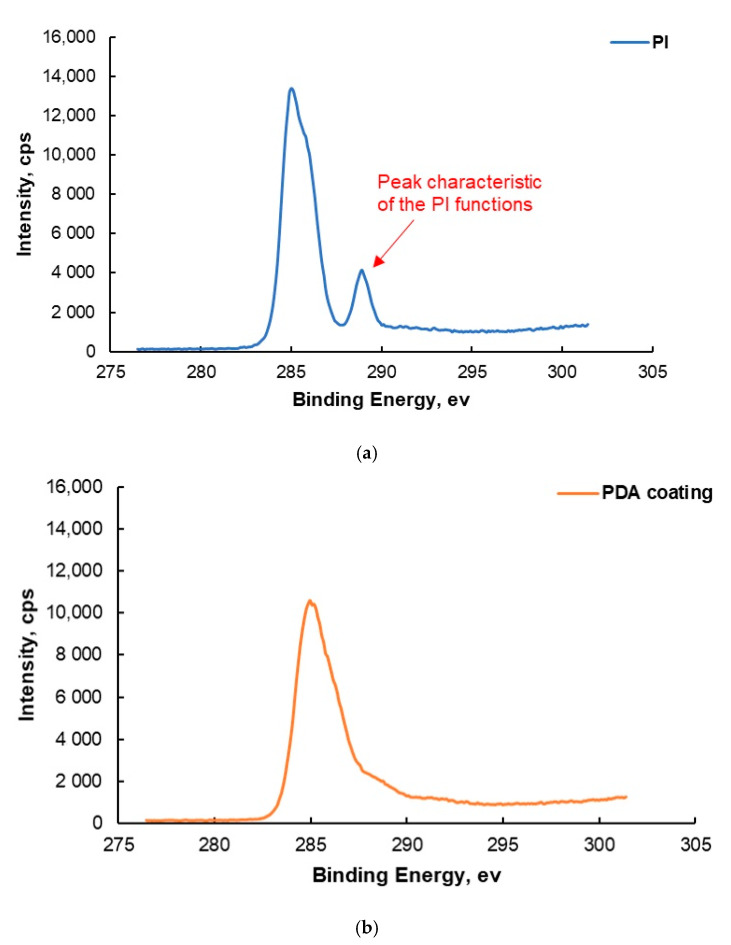
XPS spectra (C 1s high-resolution spectra) of the (**a**) uncoated PI film and (**b**) PDA-coated PI film.

The XPS measurements were carried out to detect the presence of PDA deposited onto the 50 µm-thick PI film. The resulting elemental composition of the analyzed surface as an atomic percentage is depicted in Table 6. Ca, F, and Si were detected on the survey spectra, but their amounts were not significant because the values were close to the detection limit of XPS (<0.5%). These elements were certainly coming from surface contamination. The N/C and O/C ratios were close to the stoichiometric values of polydopamine (i.e., 0.125 and 0.25, respectively) [31,32]. The XPS elemental composition of the cleaned PI film as an atomic percentage is presented in Table 7. A comparison of the elemental composition of the PDA coating (Table 6) to the cleaned PI surface (Table 7) did not give evidence of the presence of a PDA coating. Therefore, the XPS high-resolution C 1s spectra of the PI film coated with PDA and the uncoated PI film are shown in Figure 6. A comparison of the two experimental spectra reveals that the imide group peak was no longer observed after PDA coating deposition. This means the whole PI film was covered with PDA, and PI was no longer detected by XPS after the deposition of PDA, showing the deposition of a coating with a thickness of 10 nm or more (assuming an analysis depth of about 10 nm). This finding certainly shows that the PDA coating was homogeneous at the scale of the XPS scans (700 µm × 300 µm). Therefore, it was assumed that a homogeneous PDA aggregate morphology, as revealed by AFM (Figure 5c,d) should be observed at the same sub-micron scale.

### 7.5. Adhesion Mechanisms

To interpret the adhesion measurement results reported in Section 7.2, it is important to consider the adhesion theories. The most important theories of adhesion are (1) adsorption or chemical bonding, (2) mechanical adhesion, (3) diffusion, and (4) electrostatic theory. Nowadays, it is recognized that the mechanisms characteristic of these theories are difficult to consider separately [38].

The adsorption theory implies that when two materials come into close contact on a molecular scale—one in a liquid form and the other in solid form with a smooth surface—there will be forces of attraction between them [38]. The primary (or strong) chemical bonds are responsible for chemical bonding [39], and they are generally ionic, covalent, or metallic bonds. The secondary (or weak) bonds come from dipole interactions and are described as van der Waals (Debye induction, Keesom orientation, and London dispersion) or hydrogen bonds [40]. Since any atomic or molecular species has electrons, there will always, at least, be London dispersion forces (as part of van der Waals forces) that cause physical adsorption [38]. This could be the reason why, in all cases in the current study, the peel strength was larger than zero (Figure 3). For the case of cleaning (cleaning + drying) and plasma, no significant improvement in the peel strength was recorded, even though the surface chemistry of the PI was modified compared with that of the as-received PI. This shows that the bonding linked to physical adsorption did not lead to strong adhesion.

The mechanical theory claims that rough or porous surfaces provide improved adhesion compared with a case where the matching surfaces are smooth. The adhesive strength comes from the mechanical interlocking [38]. According to the mechanical theory, the higher adhesion in the case of the ground bilayer sample was due to the penetration of molten PLA into asperities of the ground PI solid surface during compression molding at 200 °C, leading to a higher bond strength while peeling the PI from the PLA substrate. The high roughness of the ground PI was recorded in AFM large-scale pictures, showing a 60-fold increase in the roughness average amplitude compared with the reference sample. However, it is important to mention here that our abrasion procedure would require automatization for better control of the surface roughness.

According to the diffusion theory, which is more broadly accepted for the same polymer (autohesion) or between very similar polymers, when two polymers are brought into contact above their glass transition temperature (Tg), the polymer chains can interdiffuse, causing the interface to fade and eventually vanish [38]. In the current study, it was assumed that interdiffusion between the two polymers could be neglected, since the two polymers were not compatible and, moreover, because the compression molding was conducted at 200 °C, which is below the glass transition temperature of polyimides (in general, above 350 °C [41,42]).

The case of PDA coating requires a thorough analysis of the results in terms of adhesion. First, as predicted by the Hansen solubility parameters (Table 3), DA was more compatible with PLA compared with PI, and hence, more chemical interaction at the interface was present when the PLA was brought into contact above its Tg with the thin PDA layer adhered on the PI surface, probably leading to a higher peel strength. Then, the change in surface chemistry between the bare PI and PI coated with PDA can be analyzed from Table 6 and Table 7. The presence of polar atoms at the surface did not change significantly between these two cases, which might explain why the polar component was not significantly modified in Figure 2d. Differences in terms of roughness were also small between the bare PI and PDA coating. A significant increase was observed between the roughness amplitudes for the 10 mm × 10 mm images from 4.04 to 6.98 nm after PDA coating. Still, the absolute difference of the values was quite small, being circa 3 nm, and the standard deviation was relatively high for these two measurements. In the end, this small difference in roughness probably explained why the surface energy values were close between the PDA coating and bare PI. However, an important limitation of the current PDA coating deposition methodology is the treatment duration (24 h), which may limit its industrial implementation as it generally requires fast treatments.

Regarding chemical etching, it was observed that both chemical etchings did not lead to any improvement in peel strength. For the HCl etching, it was also observed that the wettability was not significantly changed, which certainly means that the acid etching led to no significant surface modification of PI. In the case of NaOH etching, the treatment was expected to lead to a hydrophilic surface chemistry, with only a slight modification to the surface topography [43]. This is consistent with the present results; however, it was assumed that no increase in peel strength was observed in this case because of poor chemical compatibility between the modified PI and PLA.

**Figure 7:**
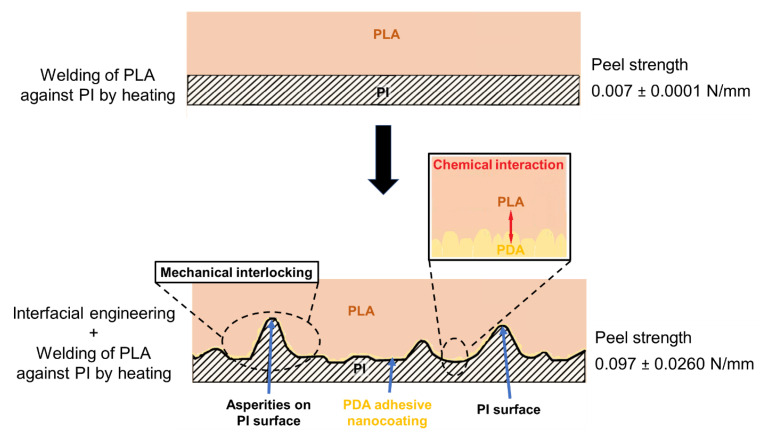
Schematic of the expected adhesion mechanisms between PI and PLA in the case of the treatment providing the highest adhesion force, which was PI surface abrasion followed by PDA coating.

To sum up, the adhesion between PI and PLA was mainly due to the interactions related to the Van der Waals bonds (cases with no PDA), chemical interaction between the compatible surfaces for the interface between PLA and PDA-coated PI, and the presence of asperities after mechanical abrasion of PI promoting adhesion through mechanical interlocking. A schematic of the expected adhesion mechanisms is shown in Figure 7 in the case providing the highest adhesion force (mechanical abrasion of PI followed by PDA coating of PI and welding of PLA against PI by heating).

## 8. Conclusions

The influence of different surface treatments on the adhesion between polyimide (PI) and polylactide (PLA), two incompatible polymers, has been studied, and the adhesion mechanisms of the most promising surface treatments have been discussed. In all the cases, PLA was welded against PI by heating prior to 90-degree peel testing. It was observed that the highest peel strength between PI and PLA could be achieved by the combination of mechanical abrasion and the use of PDA as an adhesive nanocoating (increasing from 0.007 ± 0.0001 N/mm for the untreated case to 0.097 ± 0.0260 N/mm in the case of grinding + cleaning + drying + PDA coating). Roughness plays a critical role in PI-PLA adhesion by promoting the penetration of molten PLA into the asperities of the ground PI solid surface, with this mechanism being named mechanical interlocking. Indeed, the PI film roughness was the highest in the case of grinding + cleaning + drying + PDA coating (i.e., 340 ± 170 nm for a 10 µm × 10 µm scan size), whereas the roughness of the untreated case was 4.04 ± 0.73 nm for the same scan size. PDA increased the chemical interaction and acted as a compatibilization layer between the PI and PLA, as proven by the measurement of the adhesion force (increased from 0.007 ± 0.0001 N/mm to 0.025 ± 0.0069 N/mm after PDA coating). A theoretical calculation of the materials’ compatibility was performed with the Hansen solubility parameters approach. The calculated values were consistent with the experimental results (i.e., the RED number of PLA-PI being 1.05 was substituted with the ones of DA-PI 0.58 and DA-PI 0.60, which were lower values characteristic of enhanced compatibility).

The process presented in this study to bond PI with PLA has space for further improvement. For example, the abrasion treatment was not perfectly controlled because it was performed manually. This treatment could be conducted automatically, engendering controlled patterns and roughness values. Concerning the PDA adhesive nanocoating, its deposition time (24 h) was particularly long for any potential industrial implementation, requiring a drastic decrease in the duration of this treatment. Nevertheless, the developed interfacial methodologies are ready to be implemented for the integration of an FBG sensor in an FFF-printed polymer structure.

## Figures and Tables

**Table 2:** Calculated Hansen solubility parameters of polyimide (PI) and polylactide (PLA) by means of HSPiP software.

Parameter		δ_D_	δ_P_	δ_H_	R_a_	RED PI-PLA	R_o PLA_
Unit		(MJ·m^−3^)^0.5^	(MJ·m^−3^)^0.5^	(MJ·m^−3^)^0.5^	(MJ·m^−3^)^0.5^	-	(MJ·m^−3^)^0.5^
Substance	PI	21.8	4.4	3.8	11.3	1.05	10.7
	PLA	17.7	8.7	10.2			

**Table 3:** Calculated Hansen solubility parameters of dopamine (DA).

Parameter		δ_D_	δ_P_	δ_H_	R_a_ DA–PI	RED DA–PI	R_a_ DA–PLA	RED DA–PLA	R_o PI_
Unit		(MJ·m^−3^)^0.5^	(MJ·m^−3^)^0.5^	(MJ·m^−3^)^0.5^	(MJ·m^−3^)^0.5^	-	(MJ·m^−3^)^0.5^	-	(MJ·m^−3^)^0.5^
Substance	DA	19.6	7.6	15.2	12.6	0.58	6.4	0.60	21.6

**Table 4:** The six investigated cases and related hypotheses on adhesion.

Case	Hypothesis to Increase Adhesion			
	Wettability Increase by Cleaning	Mechanical Interlocking by Increasing Roughness	Wettability Increase by Chemical Modification of the Surface	Chemical Interaction Increase by Means of a Coating
cleaning + drying	×			
grinding + cleaning + drying	×	×		
cleaning + drying + PDA coating	×			×
cleaning + drying + plasma	×		×	
cleaning + drying + chemical etching	×	×	×	
grinding + cleaning + drying + PDA coating	×	×		×

**Table 5:** The Sa roughness values of the PI-PLA samples exhibiting the highest adhesion force, including the reference sample after cleaning and drying.

Surface Treatment	Scan Size	
	1 µm × 1 µm	10 µm × 10 µm
cleaning + drying	1.98 ± 0.94 nm	4.04 ± 0.73 nm
cleaning + drying + PDA coating	1.85 ± 0.70 nm	6.95 ± 1.98 nm
grinding + cleaning + drying	6.85 ± 2.57 nm	240 ± 120 nm
grinding + cleaning + drying + PDA coating	8.00 ± 6.52 nm	340 ± 170 nm

**Table 6:** XPS elemental composition of PDA coating as an atomic percentage.

Composition (%)						
	C 1s %	Ca 2p %	F 1s %	N 1s %	O 1s %	Si 2p %
Experimental	74.9	0.3	0.1	6.6	18.0	0.2
Theoretical	72.7	-	-	9.1	18.2	-

**Table 7:** XPS elemental composition of cleaned PI film as an atomic percentage.

Composition (%)								
	C 1s %	Ca 2p %	N 1s %	Na 1s %	O 1s %	Si 2p %	Cl 2p %	I 3d %
Experimental	78.3	0.3	5.1	0.3	15.6	0.2	0.1	0.1
Theoretical	75.9	-	6.9	-	17.2	-	-	-

## Data Availability

The data presented in this study are available on request from the corresponding author.

## Abbreviations

AFM	atomic force microscopy
CA	contact angle
DA	dopamine
DBD	dielectric barrier discharge
FBG	fiber Bragg grating
FFF	fused filament fabrication
HCl	hydrochloric acid
HSP	Hansen solubility parameters
HSPiP	Hansen solubility parameters in practice
NaOH	sodium hydroxide
OWRK	Owens, Wendt, Rabel, and Kaelble
PDA	polydopamine
PI	polyimide
PLA	poly(lactic acid)
R_a_	spatial distance between the HSPs of the two considered substances
RED	ratio between R_a_ and R_o_ defining the relative energy difference of the system
R_o_	sphere radius in the case of the substance PLA or PI
Sa	average roughness amplitude
SE	surface free energy
SMILES	simplified molecular input line entry specification
Tg	glass transition temperature
XPS	x-ray photoelectron spectroscopy
δ_D X_	cohesion energy from the dispersion forces calculated for the substance X (X = PLA, PI, or DA)
δ_H X_	cohesion energy from the hydrogen bonding calculated for the substance X (X = PLA, PI, or DA)
δ_P X_	cohesion energy from the polar forces calculated for the substance X (X = PLA, PI, or DA)
